# Exploring the environmental drivers of waterfowl movement in arid landscapes using first-passage time analysis

**DOI:** 10.1186/s40462-016-0073-x

**Published:** 2016-04-01

**Authors:** Dominic A. W. Henry, Judith M. Ament, Graeme S. Cumming

**Affiliations:** Percy FitzPatrick Institute, DST/NRF Centre of Excellence, University of Cape Town, Rondebosch, Cape Town 7701 South Africa; Centre for Biodiversity and Environment Research, Department of Genetics, Evolution and Environment, University College London, London, WC1E 6BT UK; Institute of Zoology, Zoological Society of London, Regent’s Park, London, NW1 4RY UK; ARC Centre of Excellence for Coral Reef Studies, James Cook University, Townsville, QLD 4811 Australia

**Keywords:** Waterfowl, NDVI, Rainfall, Southern Africa, Nomadic, First-passage time, *Alopochen aegyptiaca*, *Anas erythrorhyncha*

## Abstract

**Background:**

The movement patterns of many southern African waterfowl are typified by nomadism, which is thought to be a response to unpredictable changes in resource distributions. Nomadism and the related movement choices that waterfowl make in arid environments are, however, poorly understood. Tracking multiple individuals across wide spatiotemporal gradients offers one approach to elucidating the cues and mechanisms underpinning movement decisions. We used first-passage time (FPT) to analyse high spatial and temporal resolution telemetry data for Red-billed Teal and Egyptian Geese across a 1500 km geographical gradient between 2008 and 2014. We tested the importance of several environmental variables in structuring movement patterns, focusing on two competing hypotheses: (1) whether movements are driven by resource conditions during the current period of habitat occupation (reactive movement hypothesis), or (2) whether movements are structured by shifts in the magnitude and direction of environmental variables at locations prior to occupation (prescient movement hypothesis).

**Results:**

An increase in rainfall at a 32 day lag (i.e., prior to wetland occupancy), along with tagging site, were significant predictors of FPT in both waterfowl species. There was a positive relationship between NDVI and FPT for Egyptian Geese during this 32 day period; the relationship was negative for Red-billed Teal. Consistent with findings for migratory grazing geese, Egyptian Geese prioritised food quality over food biomass. Red-billed Teal showed few immediate responses to wetland filling, contrary to what one would predict for a dabbling duck, suggesting high dietary flexibility. Our results were consistent with the prescient movement hypothesis.

**Conclusions:**

Using FPT analysis we showed that the proximate drivers of southern African waterfowl movement are the dynamics of rainfall and primary productivity. Waterfowl appeared to be able to perceive and respond to temporal shifts in resource conditions prior to habitat patch occupation. This in turn suggests that their movements in semi-arid landscapes may be underpinned by intimate knowledge of the local environment; waterfowl pursue a complex behavioural strategy, locating suitable habitat patches proactively, rather than acting as passive respondents.

## Background

Processes that drive movement occur on a wide range of spatiotemporal scales and are important for the structure and dynamics of populations, communities and ecosystems [1, 2]. In order to adequately link movement patterns and changes in landscape conditions it is necessary to track multiple individuals across broad geographic and seasonal gradients, while simultaneously accurately quantifying the dynamics of landscape resources of interest [3]. The development of lightweight tracking devices with the ability to record high resolution movement data, coupled with broad scale remote sensing data has in many cases made this possible [4–6]. However, an important challenge lies in detecting phases of movement within the complete path, as well as revealing the environmental factors that drive the emergence and persistence of these phases [7].

Under certain circumstances theory suggests that animals should move slowly and tortuously through habitats containing high quality resources [8] — a behaviour analogous to Area-Restricted Search (ARS; [9]). Patches in which movement is tortuous should be profitable habitats which provide adequate resources that increase fitness through energy acquisition, reproduction and survival. Animals should avoid areas that have negative fitness consequences by moving more quickly and linearly through them. Identifying landscape characteristics in which movements are clustered can provide insight into the factors that shape an animal’s movement through a landscape. It is important to note that these movement patterns can be confounded by several factors which may obscure the relationship between environmental resources and habitat use – including the high levels of individual variation in animal movements; competition; predation; social factors and life-stage requirements.

Resources in most ecosystems are heterogeneously distributed across space and through time [10]. They are organised within a scale-dependant hierarchy, with aggregations (patches) at smaller scales nesting into those at larger scales [11, 12]. The density and dynamics of available resources therefore depend on the scale(s) at which an animal interacts with the landscape [13]. To maximise fitness, mobile animals should be able to alter their behaviour to exploit resources at different scales [8]. Animal movement is a potentially vital mechanism for dealing with heterogeneous landscapes and movement patterns should therefore provide evidence for spatial responses [14].

The presence of water is an essential habitat resource for all waterfowl. Wetlands in semi-arid landscapes are dynamic entities and are usually in a state of flux. The landscape that southern African waterbirds inhabit is generally arid, with unpredictable timing and duration of rainfall events [15]. The dynamics of filling and drying cycles are primarily driven by the stochastic nature of rainfall events. In southern Africa dry periods are common and can last years, but these can be followed by unpredictable periods of above average rainfall [16]. This creates a spatially and temporally variable mosaic of ephemeral wetlands. Many waterbirds occurring in such areas have adapted to this variability by employing nomadic movements during parts of the year [17–19]. Egyptian Geese *Alopochen aegyptiaca* and Red-billed Teal *Anas erythrorhyncha* are two species of southern African waterfowl that adopt widespread nomadic movements [20]. This makes them ideal study species for investigating ARS behaviour in response to environmental heterogeneity.

If waterfowl perform broad scale movements and adopt ARS behaviour opportunistically when suitable resources are encountered, then variation in first-passage time should be best explained by resource conditions in the period during which waterfowl occupy a given area – termed here as the “reactive movement (RM)” hypothesis. This would suggest that movement decisions are a response to current local and immediate environmental conditions. This hypothesis implies the following two predictions: RM_1_) If forage availability is an important environmental driver of movements, FPT would be positively correlated with local food biomass, measured by the normalised difference vegetation index (NDVI; [21]). Egyptian Geese are primarily grazers and thus are reliant on vegetation which may surround wetlands, while Red-Billed Teal are dabbling ducks and so rely on food resources located within the water column. We would thus expect the effect of vegetation greenness (NDVI) to be stronger for geese than for teal. RM_2_) Wetlands are a primary abiotic resource for waterfowl and provide habitats for foraging, roosting, safety from predators, and moult sites. If the extent of a wetland is an important environmental driver of movements, FPT would be positively correlated with either rainfall or wetland area or a combination of both. Many ephemeral wetlands in southern Africa are shallow rain-fed depressions in which inundation, and hence wetland area, is closely tied to local precipitation events. However, an increase in wetland area is not necessarily associated with higher local rainfall. For example, floodplains can inundate following rainfall events in more distant regions of the catchment basin.

Alternatively, waterfowl may be able to structure their movements in response to changes in the magnitude and direction of resources states leading up to habitat patch occupation. There are a number of potential mechanisms that may drive this behaviour. For instance, well developed spatial memory of the landscape coupled with ability to incorporate information about local weather conditions could allow waterfowl to make movement decisions which are distinctly different to those described in the RM hypothesis. Under this hypothesis, FPT would be best explained by shifts in the magnitude and direction of resource states between the current time of occupation and a lag period prior to bird arrival - here termed the “prescient movement (PM)” hypothesis. We investigated the hypothesis with both 16 and 32 day lag periods. A positive increase in NDVI between two time periods, which reflects changes in vegetative growth, indicates an increase in food quality [22]. It has been found that younger plants have higher nutritional quality (higher nitrogen concentration) and lower levels of secondary plant chemicals. Following the Green Wave Hypothesis (GWH) it has been demonstrated that northern hemisphere geese do not select habitats with the highest biomass, but instead time their migration to take advantage of successive peaks of plant nutrition and digestibility [23, 24]. Our second hypothesis implies the following two predictions: PM_1_) If waterfowl movements are a response to food quality, we would expect FPT to be higher in areas that experienced a positive change in NDVI in the 16 or 32 days prior to occupation of a patch. We would expect this effect to be more important for geese, which are grazers, than for teal, which are traditionally thought to be more reliant on invertebrate and macrophyte food resources. If support for the PM hypothesis emerged we also expected that (PM_2_) the first-passage time of Red-billed Teal should be longer in areas that experienced positive changes in wetland cover and/or rainfall prior to bird arrival. For Egyptian Geese we also predicted a positive correlation between FPT and increases in rainfall and wetland area, but we expected this response to be more prominent at the 32 day lag period at which water levels start to recede and vegetation starts to grow on previously submerged shorelines.

These predictions require some additional explanation. Wetlands are dynamic entities and in many cases are either filling or drying down. These two states represent different opportunities for Egyptian Geese and Red-billed Teal, and a successional response by waterbirds to rainfall events and wetland filling has been demonstrated in arid zone systems [25, 26]. In Australia, Kingsford et al. [25] found that dabbling ducks arrive first to take advantage of the boom period, when nutrients are mobilised and dormant invertebrates emerge and reproduce. Grazing birds, conversely, have a lagged response to rainfall events and may arrive as wetlands start to dry down, utilizing terrestrial plants that colonize the drying shorelines [25]. Studies in southern Africa have recorded Red-billed Teal arriving at inundated wetlands within days of rainfall events, with numbers peaking after a couple of weeks [27, 28]. Large variation in response time appears to exist (e.g., Red-billed Teal abundance peaked 4 months following the inundation of a large river system in Namibia, [26]).

We addressed the interaction between external factors, characterised by landscape attributes, and the navigation capacity of two species of southern African waterfowl. Navigation capacity describes the ability of organisms to decide when and where to move. Effective navigation requires the ability to detect and respond to the spatial and temporal dynamics of underlying environmental conditions [7]. We first used FPT analysis to determine the scale of movement of waterfowl over yearly temporal scales across a 1500 km geographical gradient. We then explored the spatiotemporal dynamics and relative importance of abiotic and biotic variables associated with habitat resources required by waterfowl. We aimed to identify key environmental variables that influence movement behaviour.

## Methods

### Sites and study populations

The birds in our study population were captured at three wetland sites in South Africa and one in Zimbabwe: Strandfontein wastewater treatment works (34°05′ S, 18°20′ E); Barberspan Nature Reserve (26°33′ S, 25°37′ E); Jozini Dam (27°20′ S, 31°54′ E) and Lake Manyame (17°49′ S, 30°36′ E), respectively (see Appendices 1 and 2 for capture sites and movement paths of all individuals). Strandfontein experiences a Mediterranean climate with the majority of rainfall occurring in the winter months. Barberspan, Jozini Dam and Lake Manyame fall within summer rainfall areas, although the timing and amount of precipitation is highly variable (see [29] for further details of sites). Semi-arid conditions are common over most of southern Africa; mean rainfall over the entire region is 475 mm.

### Movement data

The telemetry data were derived from Egyptian Geese and Red-billed Teal tagged with satellite GPS platform transmitter terminals (30 and 22 g PTTs respectively; Microwave Telemetry Inc., Columbia, MD, USA). PTTs were set to record a GPS location every 2 h for geese and 4 h for teal (for details of transmitter attachment methods and success rates, see [30]). Birds were tagged immediately after they had completed moult, which allowed us to confirm the wetlands as moulting sites. Birds tracked for less than 90 days were excluded from the analysis. The resulting sample size for Egyptian Geese was n = 19 and Red-billed Teal n = 14 (Table 1). Note that no teal were tagged at Jozini Dam and so data was only available for the three remaining populations. The duration between fixes in the tracks of each bird were inspected and tracks were split if the time between fixes was greater than 1 week (split tracks of each individual are denoted as either *a, b* or *c* dependent on the number of gaps detected – see Table 1). Note that all split tracks had a duration of greater than 90 days.

**Table 1:** Details of individual GPS-tagged Egyptian Geese (EG) and Red-billed Teal (RBT)

PTT	Spp	Site	Start	End	ND	TF	FD	NS	UDs	UDA (km2)	UDD (days)
77092	RBT	STR	3/12/2008	3/26/2009	379	1804	4.8	5	31	2.24 ± 2.42	15.8 ± 23
77093	RBT	STR	3/12/2008	9/7/2008	179	993	5.5	3	12	1.95 ± 1.24	22.1 ± 20.7
77098	RBT	STR	3/14/2008	11/24/2009	620	3550	5.7	5	44	2.26 ± 2.18	20.6 ± 30.8
77099	RBT	STR	3/14/2008	5/15/2009	427	1859	4.4	4	23	3.41 ± 3.27	20.8 ± 25.2
77100	RBT	STR	3/14/2008	4/16/2009	398	2046	5.1	6	42	1.95 ± 1.32	14.5 ± 16.9
77101	RBT	BAR	4/9/2008	9/28/2008	172	740	4.3	3	11	3.42 ± 2.19	22.2 ± 22.6
77102	RBT	BAR	4/10/2008	4/20/2010	740	4155	5.6	4	82	1.54 ± 0.98	13.5 ± 24
77103	RBT	MAN	5/5/2008	8/24/2008	111	610	5.5	3	33	2.57 ± 2.73	6 ± 7.6
77104	RBT	MAN	5/5/2008	1/25/2009	265	1431	5.4	3	43	1.79 ± 1.74	10.3 ± 21.3
77106	RBT	MAN	5/6/2008	7/25/2009	445	2587	5.8	7	34	2.3 ± 2.09	19.4 ± 25.2
77108	RBT	MAN	5/6/2008	8/29/2008	115	644	5.6	4	22	2.67 ± 3.1	10.5 ± 11.9
77109	RBT	MAN	5/7/2008	12/24/2008	231	1307	5.7	4	37	1.88 ± 1.12	12.3 ± 23.2
77112	RBT	BAR	6/7/2008	5/15/2009	342	1843	5.4	3	23	2.15 ± 2.49	21.8 ± 43.6
77115	RBT	BAR	10/11/2008	7/15/2009	277	1429	5.2	4	20	2.16 ± 1.37	11.6 ± 15
77094	EG	STR	1/12/2008	5/9/2008	118	1218	10.3	4	15	1.99 ± 1.14	12.1 ± 9.6
77094a	EG	STR	8/20/2008	5/1/2009	254	2686	10.6	3	9	2.59 ± 2.29	21.2 ± 34.3
77095	EG	STR	1/12/2008	1/3/2009	357	3397	9.5	5	32	2.29 ± 1.93	15.5 ± 22.3
7711702	EG	JOZ	5/4/2012	9/20/2012	139	1669	12.0	5	13	2.25 ± 1.24	13.3 ± 13.9
7711802	EG	STR	1/17/2009	10/11/2010	632	6453	10.2	5	58	2.56 ± 2.93	18.1 ± 31.3
7712002	EG	JOZ	5/4/2012	5/24/2013	385	4317	11.2	2	86	3.12 ± 3.06	11.5 ± 17.7
7712002a	EG	JOZ	6/9/2013	1/31/2014	236	2592	11.0	3	43	1.93 ± 1.6	10.9 ± 15.9
7712102	EG	JOZ	5/5/2012	9/3/2012	121	1309	10.8	3	4	2.21 ± 1.19	30.5 ± 19.8
7712202	EG	BAR	10/23/2008	5/30/2009	219	2123	9.7	5	37	1.87 ± 1.7	8.5 ± 13.4
7712302	EG	STR	12/5/2008	6/2/2009	179	1756	9.8	4	7	2.53 ± 1.26	23.9 ± 30.7
77125	EG	MAN	5/7/2008	2/21/2010	655	6965	10.6	5	120	2.29 ± 2.98	11.6 ± 24.1
77125a	EG	MAN	4/17/2010	5/31/2011	409	3689	9.0	3	60	2.68 ± 2.76	15.6 ± 21.4
77126	EG	MAN	5/7/2008	12/26/2008	233	2682	11.5	5	29	2.33 ± 2.86	12.6 ± 16.5
77127	EG	BAR	6/7/2008	5/10/2010	702	6351	9.0	6	54	2.7 ± 3.32	17.3 ± 26.9
77128	EG	BAR	6/22/2008	6/6/2009	349	3551	10.2	3	31	1.81 ± 1.9	10.4 ± 25.4
77128a	EG	BAR	8/15/2009	5/25/2010	283	2551	9.0	3	64	1.81 ± 1.9	6.5 ± 14.3
77128b	EG	BAR	9/25/2010	5/6/2011	223	1998	9.0	3	28	1.67 ± 0.96	5.7 ± 12.1
77128c	EG	BAR	7/31/2011	12/2/2011	124	653	5.3	4	16	2.09 ± 1.4	8.8 ± 11.5
77129	EG	BAR	6/7/2008	5/15/2009	342	3491	10.2	5	61	2.13 ± 1.73	9.6 ± 17.5
77130	EG	BAR	11/9/2008	9/19/2009	314	2489	7.9	5	76	2.2 ± 1.76	8.6 ± 12.6
77130a	EG	BAR	10/4/2009	6/4/2010	243	2140	8.8	5	78	1.86 ± 1.5	6.5 ± 10.8
77132	EG	BAR	6/7/2008	5/30/2009	357	2601	7.3	3	35	2.01 ± 1.94	15.5 ± 25.4
77132a	EG	BAR	8/13/2009	4/14/2010	244	2090	8.6	2	12	2.15 ± 0.46	14.8 ± 27.3
7713301	EG	STR	12/4/2008	4/27/2009	144	1506	10.5	5	40	1.83 ± 1.2	4 ± 5.1
7713302	EG	JOZ	5/4/2012	2/19/2013	291	3009	10.3	3	27	2.81 ± 2.17	15.7 ± 23
77134	EG	STR	12/1/2008	7/29/2010	605	5330	8.8	4	19	3.28 ± 2.47	24.2 ± 28.8
77134a	EG	STR	8/19/2010	5/2/2011	256	2561	10.0	5	17	2.04 ± 2.16	19.2 ± 29.9
77134b	EG	STR	7/22/2011	4/12/2012	265	2401	9.1	3	8	2.08 ± 1.66	47.1 ± 46.2
77135	EG	STR	12/1/2008	2/8/2011	799	8522	10.7	4	107	2.5 ± 2.82	11.5 ± 18

PTT, transmitter identity; BAR, Barberspan; STR, Strandfontein; MAN, Lake Manyame; JOZ, Jozini Dam. Start and end date refers to the time period of tracking data used in the study (ND is the total tracking duration in days). Total fixes (TF) are the number of relocations recorded over the study period, while mean fixes per day (FD) are the total number of relocations divided by the number of days the transmitter was active. The remaining columns contain data from the results of the FPT analysis. NS, number of movement segments per track; UDs, number of utilisation distributions derived from all segments; UDA, mean (± sd) area of utilisation distributions; UDD, mean (± sd) number of days spent in each utilisation distribution polygon

### First-passage time analysis

**Fig. 1:**
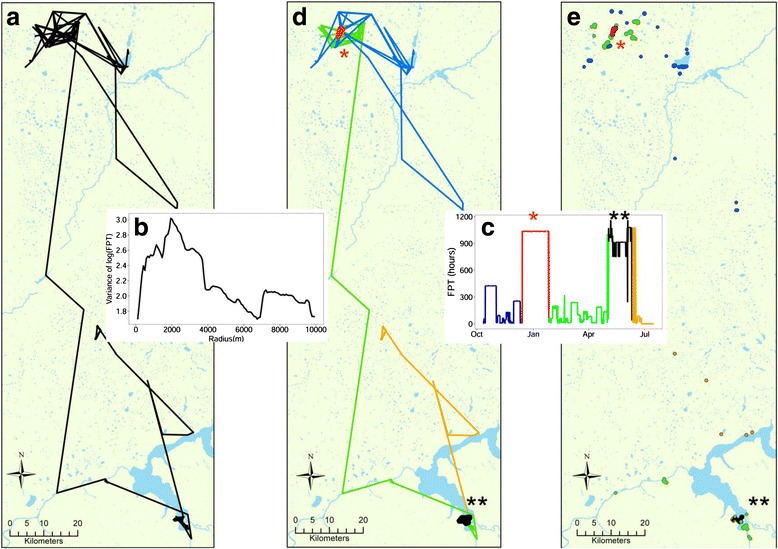
Five sequential steps which illustrate the analytical processes carried out on all birds. The movement path in this example was taken from a Red-Billed Teal (77115) tagged in Barberspan. **a**) The full movement path of the individual. **b**) The output of the first-passage time (FPT) procedure which identifies the scale at which movements are clustered. The scale corresponds to the radius at which the variance of log(FPT) is at a maximum. **c**) A graph of the magnitude of FPT at each GPS fix. The five colours each represent a movement segment identified by the Lavielle segmentation process. The red and black segments illustrate areas in which movements are highly clustered and non-linear. **d**) The initial movement path colour coded according to the corresponding segment from step c. The asterisks indicate the location of the two highly clustered movement segments (red and black). **e**) The utilisation distributions polygons derived from applying kernel density estimators to movement paths from each segment. Each polygon represents a sampling unit in the statistical analysis where mean FPT and environmental variables were measured

A graphical example of the analytical steps for the movement path of an individual bird (Red-Billed Teal 77115 from Barberspan) is shown in Fig. 1. First-passage time is calculated at each GPS fix along a movement path (Fig. 1a) as the time taken to cross a circle of a given radius [31]. The process is repeated over a range of circles with differing radii. The peaks in variance of log transformed FPT at a specific radius (Fig. 1b) indicates the scale at which an animal’s movements are clustered and hence the spatial scale at which ARS behaviour is occurring [31]. In other words, the peaks correspond to a specific circle radius in which more tortuous and intensive movements are performed. As mean FPT is increases with circle size, we applied a common radius to all bird movement paths of each species, to allow for comparisons of individual bird FPTs. For each species we used the radius at which mean variance of log FPT showed a peak. Following Le Corre, Dussault and Côté [32], FPT was calculated along an individual’s path with a given radius *r*, ranging from 100 to 10 000 m at 80 m intervals, centred on consecutive locations. The radius *r*_*max*_ is the radius at which the variance of log transformed FPT *var*_*fpt*_ reaches a maximum. The mean of variance *var*_*fpt mean*_ was calculated by averaging *var*_*fpt*_ of each bird at each radius. The peak in this mean *var*_*fpt mean*_ was then taken at a population average and used as the common spatial scale for all subsequent analysis.

Once FPT analysis was applied to each individual, plots were created of GPS fixes against FPT (Fig. 1c). Lavielle’s segmentation method was then applied in order to identify homogenous movement bouts within an individual’s movement path using the *lavielle* function in the *adehabitatLT* R package [33, 34]. The method aims to detect breakpoints in the movement path by minimizing a penalised contrast function [35]. Given that a movement path is made up of *K* segments, the method searches for an optimal number of segments *K*_*opt*_ with which to partition the movement path. There should be a clear break in the decrease of the contrast function after *K*_*opt*_, which we identified in two ways. Firstly the break and the corresponding *K*_*opt*_, was visually detected from the plot of the contrast function. Secondly, *K*_*opt*_ was automatically detected by choosing the last value of *K* at which the second derivative of the standardised contrast function is greater than a threshold *S*. Following the recommendation of Lavielle [36], *S* was set to 0.75. These methods were used in conjunction with one another to determine the number of segments for each individual’s movement path (Fig. 1c and d).

Segments from each movement path (i.e. paths from all individuals across each site) were extracted and processed in the following way: GPS fixes within segments were used to create utilisation distribution which defined an area and time over which environmental variables could be measured (Fig. 1e). Utilisation distributions were calculated with Movement-based Kernel Density Estimator (MKDE) methods [37] using the *BRB* function within the *adehabitatHR* R package [33]. In order to reduce the effects of autocorrelation inherent in the data, we calculated a mean first-passage time value at the peak radius (*mFPT*_*Rmax*_) derived from all GPS fixes contained within the home-range polygon. If the home range was made up of multiple polygons, as is common when using MKDE methods, *mFPT*_*Rmax*_ was calculated for each polygon individually. Each polygon was then used as a sampling unit in which *mFPT*_*Rmax*_ was the response variable and the set of environmental variables measured in that polygon were the explanatory variables (Fig. 1e).

### Environmental data

In order to evaluate the relationship between FPT and environmental conditions, 12 variables were calculated for each home range polygon for each bird. Extraction of environmental variables was performed using Google Earth Engine (https://earthengine.google.com/), a cloud platform for the analysis of geospatial data. NDVI, rainfall, water surface coverage (modified normalised difference water index; mNDWI), elevation and temperature were calculated as mean values over the time *t* for which the home range was occupied (Table 2). Two extra sets of NDVI, rainfall and mNDWI variables were calculated: 1) the difference between mean at time *t* and *t-16* days and 2) the difference between mean at time *t* and *t-32 days.* A positive change in NDVI between time periods would indicate and increase in vegetative growth and hence food quality, while a positive change in mNDWI would indicate an increase in wetland extent. Time lags were chosen to correspond with the minimum temporal resolution of the predictor variables (constrained by the resolution of NDVI which is based on 16 day composites – see Table 2 for derivations and sources of data). Dynamic variables were first averaged temporally for the duration of home range occupation, followed by spatial averaging. Geographical location of capture sites was added as a predictor variable to evaluate a study area effect on first-passage times. This included four- and three-level categorical variables for Egyptian Geese and Red-billed Teal respectively. Temperature and elevation were added as fixed effects in some candidate models to assess whether there was an effect of thermal stress on movement behaviour. We developed a set of 36 singular and multi-term candidate models to evaluate our competing hypotheses (Table 3).

**Table 2:** Details of environmental variables used as predictors in the analysis of first-passage time (FPT) of two waterfowl species in southern Africa

		Source	Units	Spatial resolution	Temporal resolution	Earth Engine Layer
NDVI	Normalised difference vegetation index NDVI = (NIR – R)/(NIR + R)	MODIS terra		250 m	16-day composite	MODIS/MCD43A4_NDVI
mNDWI	Modified normalised difference water index mNDWI = (G-MIR)/(G + MIR)	MODIS terra		250 m	16-day composite	MODIS/MCD43A4
Precip	Rainfall	TRMM	mm/h	0.25°	3 hourly	TRMM/3B42
Temp	Land surface temperature	MODIS	Kelvin (Convert to °C)	1 km	Daily	MODIS/MYD11A1
Elev	Elevation	NASA	Meters above sea level	0.9 km	N/A	CGIAR/SRTM90_V4

**Table 3:** Candidate set of generalised linear mixed models used to investigate the relationship between mean first-passage time (mFPT_Rmax_) and environmental variables of Egyptian Geese and Red-billed Teal. Individual birds (ID) were added as a random effect to all models. See Table 2 and text in *Methods* for derivation of environmental predictor variables

Model			Model formula
1		Food quantity	mFPT_Rmax_ ~ NDVI_t_ + (1|ID)
2		Food quality (16 day)	mFPT_Rmax_ ~ ΔNDVI _t-16_ + (1|ID)
3		Food quality (32 day)	mFPT_Rmax_ ~ ΔNDVI _t-32_ + (1|ID)
4		Wetland cover	mFPT_Rmax_ ~ mNDWI_t_ + (1|ID)
5		Wetland cover change (16 day)	mFPT_Rmax_ ~ ΔmNDWI_t-16_ + (1|ID)
6		Wetland cover change (32 day)	mFPT_Rmax_ ~ ΔmNDWI _t-32_ + (1|ID)
7		Precipitation	mFPT_Rmax_ ~ Precip_t_ + (1|ID)
8		Precipitation (16 day)	mFPT_Rmax_ ~ ΔPrecip_t-16_ + (1|ID)
9		Precipitation (32 day)	mFPT_Rmax_ ~ ΔPrecip_t-32_ + (1|ID)
10		Site	mFPT_Rmax_ ~ site + (1|ID)
11		Temperature & elevation	mFPT_Rmax_ ~ Temp + Elev + (1|ID)
RM hypothesis:^a^			
12 (16)		Food quantity, wetland cover & precipitation (+ temperature & elevation, site)	mFPT_Rmax_ ~ NDVI_t_ + mNDWI_t_ + Precip_t_ + (1|ID)
13 (17)		Food quantity & wetland cover (+ temperature & elevation, site)	mFPT_Rmax_ ~ NDVI_t_ + mNDWI_t_ + (1|ID)
14 (18)		Food quantity & precipitation (+ temperature & elevation, site)	mFPT_Rmax_ ~ NDVI_t_ + Precip_t_ + (1|ID)
15 (19)		Wetland cover & precipitation (+ temperature & elevation, site)	mFPT_Rmax_ ~ mNDWI_t_ + Precip_t_ + (1|ID)
PM hypothesis:^b^			
20 (24)	16 day Lag	Change in food quality, wetland cover & precipitation (+ site)	mFPT_Rmax_ ~ ΔNDVI _t-16_ + ΔmNDWI _t-16_ + ΔPrecip_t-16_ + (1|ID)
21 (25)		Change in food quality & wetland cover (+ site)	mFPT_Rmax_ ~ ΔNDVI _t-16_ + ΔmNDWI _t-16_ + (1|ID)
22 (26)		Change in food quality & precipitation (+ site)	mFPT_Rmax_ ~ ΔNDVI _t-16_ + ΔPrecip_t-16_ + (1|ID)
23 (27)		Change in wetland cover change & precipitation (+ site)	mFPT_Rmax_ ~ ΔmNDWI _t-16_ + ΔPrecip_t-16_ + (1|ID)
28 (32)	32 day Lag	Change in food quality, wetland cover & precipitation (+ site)	mFPT_Rmax_ ~ ΔNDVI _t-32_ + ΔmNDWI _t-32_ + ΔPrecip_t-32_ + (1|ID)
29 (33)		Change in food quality & wetland cover (+ site)	mFPT_Rmax_ ~ ΔNDVI _t-32_ + ΔmNDWI _t-32_ + (1|ID)
30 (34)		Change in food quality & precipitation (+ site)	mFPT_Rmax_ ~ ΔNDVI _t-32_ + ΔPrecip_t-32_ + (1|ID)
31 (35)		Change in wetland cover change & precipitation (+ site)	mFPT_Rmax_ ~ ΔmNDWI _t-32_ + ΔPrecip_t-32_ + (1|ID)
36		Null	mFPT_Rmax_ ~ 1 + (1|ID)

*RM* reactive movement; *PM* prescient movement

^a^Models numbers in parentheses comprise of the same set of environmental predictors with extra addition of site, temp and elevation as a predictor variables

^b^Models numbers in parentheses comprise of the same set of environmental predictors with extra addition of site as a predictor variable

### Statistical analyses

Generalised linear mixed models were used to model the relationship between *mFPT*_*Rmax*_ and environmental variables using the *lmer* function from the R package *lme4* [38]. Data were first screened for normality, and outliers were removed. *mFPT*_*Rmax*_ was log-transformed prior to inclusion into candidate models. Individual birds were added as random effect to allow for estimation of population level regression coefficients while accounting for variation between individuals. All predictor variables were scaled before inclusion into the models. This allowed for standardisation of parameter estimates and comparison of their magnitudes. Spatial auto-correlation in the residuals of the chosen models was examined in two ways. First, we visually examined spatial plots of the magnitude and signs of residuals; and second, we used semi-variograms to quantify variance as a function of distance between points (*bubble* and *variogram* function from *gstat* R package, [39]). Model selection followed evaluation of candidate models from AIC criteria [40]. Variance inflation factors were used to test for the presence of collinearity amongst predictor variables. R^2^_GLMM_ was used as a measure of overall fit for the selected models [41].

## Results

**Fig. 2:**
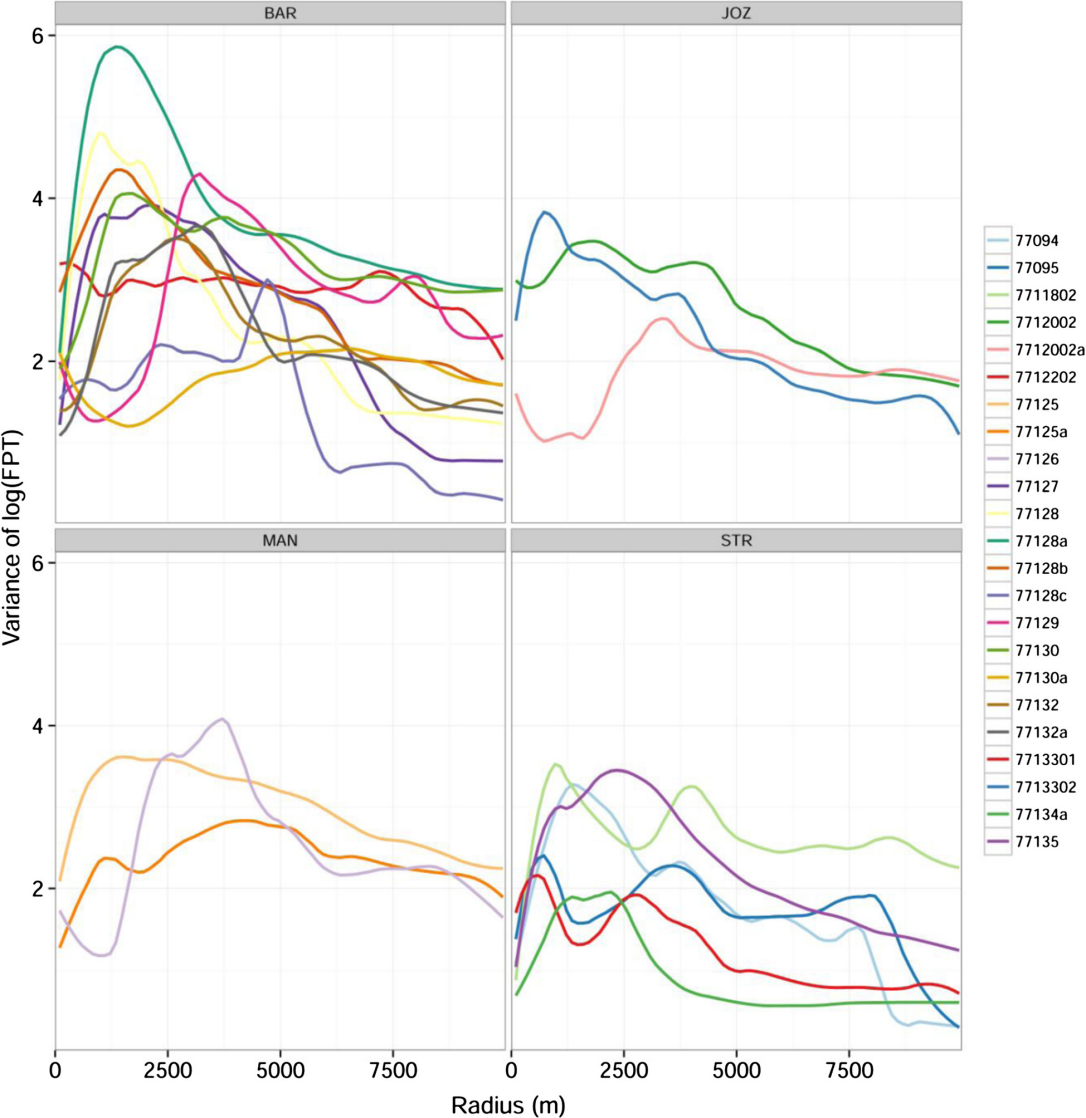
Curves of individual Egyptian Geese showing the variance in log first-passage time against circle radius. Panels correspond to individuals tagged at 4 different wetland sites. PTT, transmitter identity; BAR, Barberspan; STR, Strandfontein; MAN, Lake Manyame; JOZ, Jozini Dam

**Fig. 3:**
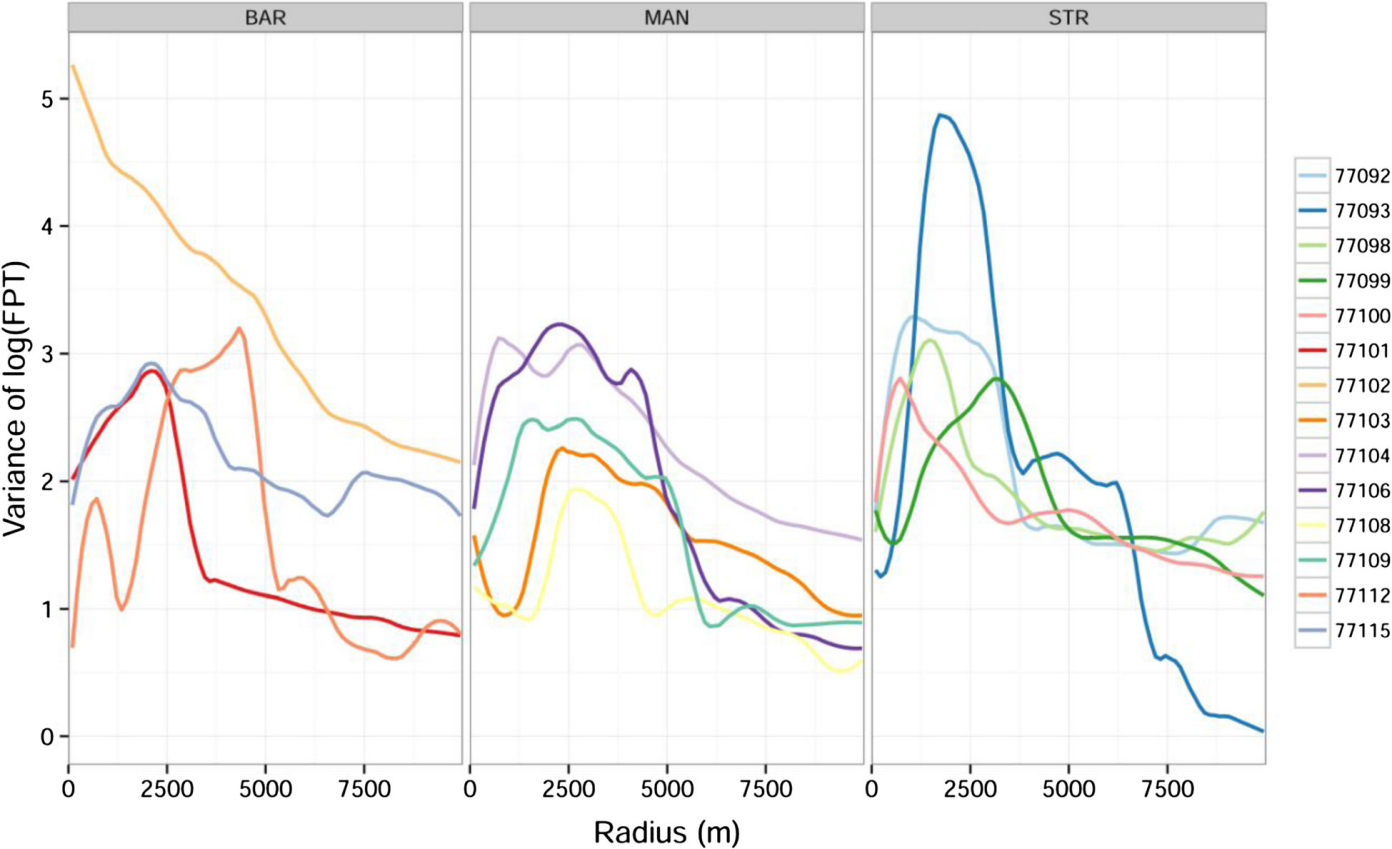
Curves of individual Red-Billed Teal showing the variance in log first-passage time against circle radius. Panels correspond to individuals tagged at 3 different wetland sites. PTT, transmitter identity; BAR, Barberspan; STR, Strandfontein; MAN, Lake Manyame

After calculating *var*_*fpt*_ for each individual against radius (Figs. 2 and 3), the mean radius *r*_*max*_ for Egyptian Geese and Red-billed Teal were identified as 2180 and 2420 m. The results of the subsequent FPT analysis showed that the number of movement segments ranged from 2 to 7 (mean 4). The number of utilisation distribution polygons derived from the segments ranged from 4 to 120 (mean 38.2). The area of those utilisation distribution polygons ranged from 1.5 to 3.4 km^2^ (mean 2.3 km^2^). The number of days spent in a specific utilisation distribution polygon ranged from 4 to 47 (mean 15). See Table 1 for the above values of each individual.

In our analysis of environmental predictors of *mFPT*_*Rmax*_, the most parsimonious model with the highest support included difference in NDVI over a 32 day lag, difference in rainfall over a 32 day lag, and geographical location for both Egyptian Geese and Red-billed Teal (Table 4). Following the AIC criteria and model selection procedure we employed, model 34 had the greatest support and suggested that variation in *mFPT*_*Rmax*_ was best explained by changes food quality, amount of rainfall, and the geographical location of individuals. The variances explained by the fixed effects (marginal R^2^) of the chosen models were 9.4 and 11.3 %, while the variances explained by both fixed and random effects (conditional R^2^) were 17.2 and 13.1 % for Egyptian Geese and Red-billed Teal respectively (Table 5). The individual level variability was noticeably higher in Egyptian Geese (7.8 %) compared to that of Red-billed Teal (1.8 %). The candidate models representing the reactive movement hypothesis were noticeably absent from the top four and nine movement models of Egyptian Geese and Red-billed Teal respectively.

**Table 4:** Comparisons of the top models (∆AIC_c_ < 20) of first-passage time as a function environmental variables of two waterfowl species in southern Africa

Model		K	AIC_c_	ΔAIC_c_	AIC_c_ Wt	Cum Wt
	Egyptian Geese					
32	mFPT_Rmax_ ~ ΔNDVI _t-32_ + ΔmNDWI _t-32_ + ΔPrecip_t-32_ + site + (1|ID)	9	1979.25	0.00	0.43	0.43
34	mFPT_Rmax_ ~ ΔNDVI _t-32_ + ΔPrecip_t-32_ + site + (1|ID)	8	1979.39	0.14	0.40	0.83
30	mFPT_Rmax_ ~ ΔNDVI _t-32_ + ΔPrecip_t-32_ + (1|ID)	5	1982.95	3.70	0.07	0.90
28	mFPT_Rmax_ ~ ΔNDVI _t-32_ + ΔmNDWI _t-32_ + ΔPrecip_t-32_ + (1|ID)	6	1983.44	4.19	0.05	0.95
19	mFPT_Rmax_ ~ mNDWI_t_ + Precip_t_ + site + (1|ID)	10	1984.52	5.27	0.03	0.98
16	mFPT_Rmax_ ~ NDVI_t_ + mNDWI_t_ + Precip_t_ + site + (1|ID)	11	1986.00	6.75	0.01	1.00
15	mFPT_Rmax_ ~ mNDWI_t_ + Precip_t_ + (1|ID)	5	1992.27	13.02	0.00	1.00
	Red-billed Teal					
34	mFPT_Rmax_ ~ ΔNDVI _t-32_ + ΔPrecip_t-32_ + site + (1|ID)	7	833.87	0.00	0.38	0.38
26	mFPT_Rmax_ ~ ΔNDVI _t-16_ + ΔPrecip_t-16_ + site + (1|ID)	7	834.79	0.92	0.24	0.62
32	mFPT_Rmax_ ~ ΔNDVI _t-32_ + ΔmNDWI _t-32_ + ΔPrecip_t-32_ + site + (1|ID)	8	835.21	1.34	0.19	0.82
24	mFPT_Rmax_ ~ ΔNDVI _t-16_ + ΔmNDWI _t-16_ + ΔPrecip_t-16_ + site + (1|ID)	8	836.83	2.95	0.09	0.90
30	mFPT_Rmax_ ~ ΔNDVI _t-32_ + ΔPrecip_t-32_ + (1|ID)	5	837.70	3.83	0.06	0.96
28	mFPT_Rmax_ ~ ΔNDVI _t-32_ + ΔmNDWI _t-32_ + ΔPrecip_t-32_ + (1|ID)	6	839.54	5.67	0.02	0.98
22	mFPT_Rmax_ ~ ΔNDVI _t-16_ + ΔPrecip_t-16_ + (1|ID)	5	840.40	6.53	0.01	0.99
20	mFPT_Rmax_ ~ ΔNDVI _t-16_ + ΔmNDWI _t-16_ + ΔPrecip_t-16_ + (1|ID)	6	842.45	8.57	0.01	1.00
35	mFPT_Rmax_ ~ ΔmNDWI _t-32_ + ΔPrecip_t-32_ + site + (1|ID)	7	852.07	18.19	0.00	1.00

Models are ranked based on differences in the corrected Akaike’s Information Criteria (ΔAIC_c_) Akaike weights (AIC_c_ Wt). K is the number of estimated parameters and Cum Wt is the cumulative weight of sequential models. Individual birds (ID) were added as a random effect to all models

**Table 5:** Summary of the generalised mixed models with the highest support in the analysis of mean first-passage time (mFPT_Rmax_) as a function of environmental variables

	Parameter	β	Lower CI	Upper CI	SE	R^2^_GLMM(m)_	R^2^_GLMM(c)_	VIF (kappa)
Egyptian Geese						9.4 %	17.2 %	3.64
	(Intercept)	1.34	1.24	1.45	0.06			
	ΔNDVI _t-32_	0.04	0.01	0.06	0.02			
	ΔPrecip_t-32_	0.12	0.09	0.14	0.02			
	Site : JOZ vs. BAR	0.04	−0.14	0.23	0.11			
	Site : MAN vs. BAR	−0.13	−0.33	0.07	0.12			
	Site : STR vs. BAR	0.25	0.10	0.40	0.09			
No. of observations: 1165, random effect groups: ID, 29								
Red-billed Teal						11.3 %	13.1 %	4.42
	(Intercept)	1.59	1.47	1.71	0.07			
	ΔNDVI _t-32_	−0.06	−0.11	−0.01	0.03			
	ΔPrecip_t-32_	0.16	0.12	0.21	0.03			
	Site : MAN vs. BAR	−0.12	−0.27	0.03	−0.11			
	Site : STR vs. BAR	0.24	0.08	0.39	0.09			
No. of observations: 445, random effect groups: ID, 14								

R^2^ values are measures of model fit based on fixed effects only (marginal variance, R^2^_GLMM(m)_) and on the full model including random effects (conditional variance, R^2^_GLMM(c)_)

**Fig. 4:**
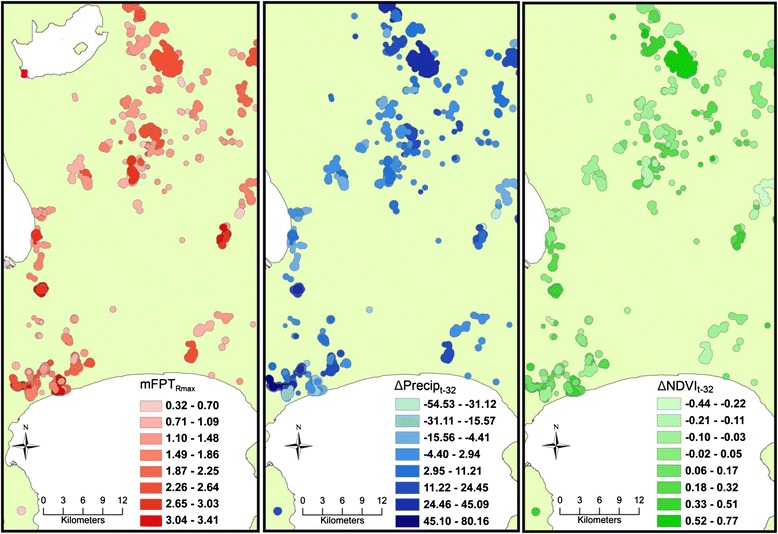
Three panels which represent a gradient of mean values of mean first-passage time (mFPT_Rmax_) and environmental variables within utilisation distribution polygons. These data illustrate the positive relationship between FPT and a 32-day lag in NDVI and precipitation (e.g. dark red, dark green and dark blue polygons represent sites at which FPT and environmental variables were strongly positively correlated). ΔNDVI_t-32_, the difference between mean NDVI within a polygon at time *t* and *t-32 days*; ΔPrecip_t-32,_ the difference between precipitation within a polygon at time *t* and *t-32 days* measured in mm. Note that mFPT_Rmax_ was measured in hours and has been logged transformed

For Egyptian Geese, differences in both NDVI and rainfall in the 32 days prior to arrival were significantly and positively correlated with *mFPT*_*Rmax*_, supporting predictions PM_1_ and PM_2_ (Fig. 4 and Table 5). The magnitude of the effect of rainfall was three times higher than that of NDVI. Individuals from the Strandfontein population had a significantly higher *mFPT*_*Rmax*_ than those of the Barberspan, which was the reference category. The parameter estimates for birds from Jozini and Lake Manyame were not significantly different from zero. For Red-billed Teal, the difference in NDVI during the 32 days prior to arrival was negatively correlated to *mFPT*_*Rmax*_ while rainfall was significantly and positively correlated with FPT (Table 5). Again the magnitude of the effect of rainfall was higher than that of NDVI. Individual teal from the Strandfontein population had a significantly higher *mFPT*_*Rmax*_ than those of the Barberspan population, while parameter estimates for birds from Lake Manyame were not significantly different from zero. We found little support for effects of temperature, elevation or mNDWI in explaining variation in FPTs There was little evidence of spatial autocorrelation in the semi-variograms and bubble plots. The kappa statistic was less than 10 for models of both species, indicating an absence of collinearity in the predictor variables.

## Discussion

Our findings show little evidence for the reactive movement hypothesis; instead, waterfowl appeared to respond to shifts in resource conditions in a given area based on changes in magnitude and direction of environmental variables between preceding lag periods and current periods of occupation. This suggests that movement decisions were potentially more complex than those that would result from randomly sampling the landscape and ceasing movement when suitable conditions were encountered. While it may be difficult to identify exactly how waterfowl perceive their landscapes, spatial awareness and prior experience may be mechanisms that might allow waterfowl to capitalise on high quality resources. Egyptian Geese spent more time in areas which had increased primary productivity and associated increases in rainfall in the 32 day period leading up to goose arrival; results which were consistent with predictions PM_1_ and PM_2_. The magnitude of the effect of rainfall was stronger than that of NDVI. By contrast, Red-billed Teal spent more time in areas in which primary productivity had decreased over the previous 32 days and where rainfall had increased, consistent with PM_2_ but not with PM_1_. Again, rainfall had a stronger effect than NDVI. Geographical location (site variable) of individuals was a significant predictor of waterfowl movement behaviour; individuals of both species had higher mean FPTs in the Strandfontein populations compared with the Barberspan populations. Temperature and elevation had no significant effect on FPT. These findings suggest both that thermoregulatory constraints do not play a role in structuring movements and that movements are not clustered in coastal regions of southern Africa. The environmental variable that represented a change in wetland extent (∆mNDWI) appeared in several of the competing models, but was not included in the final model with the highest support. This indicates that there is a potential effect of either filling or drying of wetlands on FPT, however these dynamics do not dominate the way in which waterfowl movements are structured.

The moderate amount of variance explained by the models could have resulted from several unmeasured factors affecting landscape use. For instance, waterfowl form large aggregations outside of breeding periods, and so social factors such as competition may affect the choice of habitat used. Human disturbance and predation pressure are also likely to significantly influence habitat choice and movements [42]. Another potential issue which might have influenced the explained variance is the choice of method used to delineate the geographic area over which environmental conditions were measured. We used the widely adopted movement-based KDE method which has its foundation in point-based methods. Traditional KDE methods, however, may significantly underestimate the size of utilisation distributions [43, 44]. Measuring environmental variables over a broader spatial extent would, therefore, change the calculated landscape conditions that the waterfowl would have experienced. In turn, this has the potential to alter the outcomes of the first-passage time models.

Our findings that higher FPTs of Egyptian Geese are a response to increases in primary productivity, as opposed to standing biomass, are in accordance with several studies of migratory movements of herbivorous waterfowl occurring at high latitudes in the northern hemisphere. These movements are linked to plant phenology and follow the predictions set out by the GWH, which states that waterfowl time their spring migration to take advantage of successive peaks of forage quality along their migration routes [23, 24, 45–47]. Although semi-nomadic waterfowl, living in low productivity environments where the distribution of resources is patchier [48], have different constraints in terms of locating resources to those of migrants (i.e. lack of distinct and predictable seasonal changes), they seem to prioritise forage quality in a similar manner. Responding to such changes, however, requires that waterfowl have some sort of prior knowledge of the state of landscape resources and do not simply perform random searches through the landscape to settle where conditions are suitable.

For birds living in semi-arid areas, there are trade-offs between when to stay and when to leave an area [49]. The ability of waterfowl in our study system to possess spatial awareness could allow them to capitalise on highly nutritious food sources and leave areas when nutritional quality starts to decline, providing an adaptive advantage through periods of resource uncertainty. The role of spatial memory in movement has recently received attention [50–53] and there are indeed fitness benefits of memory in heterogeneous landscapes of intermediate complexity. Waterfowl may employ a similar strategy to that of other nomadic birds (e.g., Snail Kites [54] and Pacific Black Ducks [55]) in that exploratory movements are adopted through periods of high resource abundance. This would allow waterfowl to attain a level of familiarity with high quality resource patches, avoiding the need to search extensively when resource abundance is low. Memory and prior knowledge therefore have the potential to be particularly relevant to waterfowl movement strategies in arid landscapes [56]. Indeed, in this study Egyptian Geese adopted behaviour that allowed them to respond to food quality in a similar manner to that of migrant geese following the green wave.

Kingsford et al. [25] developed a conceptual model of the movement, breeding and feeding response of five arid-zone waterbird functional groups (dabbling and diving ducks, herbivores, piscivores, large waders and small waders). They proposed that grazing and dabbling (or invertebrate) feeders should have different temporal responses to rainfall and wetland filling events. Dabbling ducks should arrive first to take advantage of invertebrates which have hatched following wetland inundation, while grazers should lag in their response to capitalise on terrestrial or aquatic plants which grow and germinate more slowly. Egyptian Geese responded to rainfall at a 32 day lag period which is what we would expect for a grazing bird feeding on emergent vegetation on freshly exposed shorelines.

Observational studies of Red-billed Teal responses to rainfall events in southern Africa support ideas that invertebrate feeders should respond quickly [27, 28]. Data from longer term studies, however, suggest that this peak in abundance occurs at a much longer lag period, with a peak occurring at 4 months post rainfall [26]. Our finding that teal responded to rainfall at a 32 day lag rather than a 16 day lag might be explained by their niche breadth. Petrie [57] showed that teal have considerable dietary flexibility. During energetically demanding periods such as the breeding season, for example, invertebrate consumption was < 14 % of total food items while the majority of remaining energy requirements was satisfied by the consumption of native grass seeds which surround wetlands. The relationship between NDVI and FPT in Red-billed Teal was opposite to that found in Egyptian Geese. First-passage time was higher in areas in which primary productivity had decreased over a 32 day lag period. It is possible that there was a higher abundance of grass seeds available as growth decreased, providing teal with an adequate food resource. These results suggest that responses to rainfall events vary considerably across apparently similar arid zone landscapes and that dietary flexibility may drive changes in movement responses between species.

One question that remains enigmatic is which cues waterfowl use to detect distant rainfall events. It has been proposed that they might be able to sense rain fronts, but the evidence that waterfowl respond to lag variables and not immediate conditions suggests that they have some knowledge of landscape conditions and can make decisions based on environmental cues [55]. There is evidence of a similar response in other species. Red-billed Quelea *Quelea quelea*, small granivorous passerines in southern Africa, are able to respond to dynamic changes in resources [58]; they appear to move ahead of rainfall events and then track back towards areas in which rain has fallen to take advantage of grass seeds.

Both Egyptian Geese and Red-billed Teal had higher than average FPTs in utilisation distributions in the Strandfontein population than in the Barberspan population. Although there was no significant difference between the other sites, Jozini had slightly higher FPT than Barberspan while the Lake Manyame population had slightly lower average FPT (Table 5). It is interesting to note that the direction and magnitude of these patterns were consistent between both these species, which indicates the important influence of landscape conditions on movement in comparison to differences in life history and ecological traits between the two species. Differing responses to environmental variation by populations of a species has been shown in several instances [59–61]. This follows a theoretical prediction that increased variation of movement responses within a species range should be associated with increased variability of resources at broad landscape scales [55, 59, 62]. This is indeed evident in our study as Barberspan and Strandfontein occur in noticeably different landscapes. Barberspan lies in an arid summer rainfall region, whereas Strandfontein is a winter rainfall region with less variability in the timing and amount of precipitation (Appendix 3). The landscape surrounding Strandfontein is characterised by a high density of grain producing agricultural land. Associated with these farms are small dams used for storage, many of which have stable water levels throughout the year. On the other hand, areas into which many of the individuals from the Barberspan population moved were more arid, with agricultural land separated by semi-deserts (Appendices 1, 2 and 3). Spatiotemporal correlation of resources was thus higher near Strandfontein and could mean there is less need for waterfowl to move long distances, resulting in higher first-passage times. This illustrates the range in strategies of nomadic movement, which is proposed to be an outcome of spatiotemporal correlation in landscape resources [63]. Differences in movement behaviour (measured by, for example, parameters such as daily movement rates, distance moved, turning angles) in these populations has previously been demonstrated [64], indicating that populations of Egyptian Geese at Barberspan and Strandfontein move in different ways, while little separated the movements of different populations of Red-billed Teal. In our analyses, however, the data showed clear differences in patterns of FPT (Figs. 2 and 3, Table 1). This suggests that analysing movements in the FTP framework can provide new as well as complementary insights into existing drivers of waterfowl movement. Additionally, it is important to recognise the variation of individuals within the same population in understanding population level processes [65, 66].

## Conclusions

We were able to undertake the first quantitative analysis of the interaction between external factors and navigational capacity of southern African waterfowl in the context of a current movement ecology framework. More generally, we have shown the utility of linking long term telemetry data over broad geographic scales with environmental conditions experienced by multiple individuals to uncover the proximate drivers of waterfowl movement. The analysis of movement using the FPT method allowed us to conclude that waterfowl movements in southern Africa are a response to the dynamics of rainfall and primary productivity. In addition, our findings suggest that waterfowl movements are not simply reactive but rather involve mechanisms which allow waterbirds to integrate information of the local landscape in order to take advantage of productive habitats. Future research should take the form of a more detailed analysis of movement and changes in resources to further understand the mechanism underlying the prescient movement hypothesis.

## Figures and Tables

**Fig. 5:**
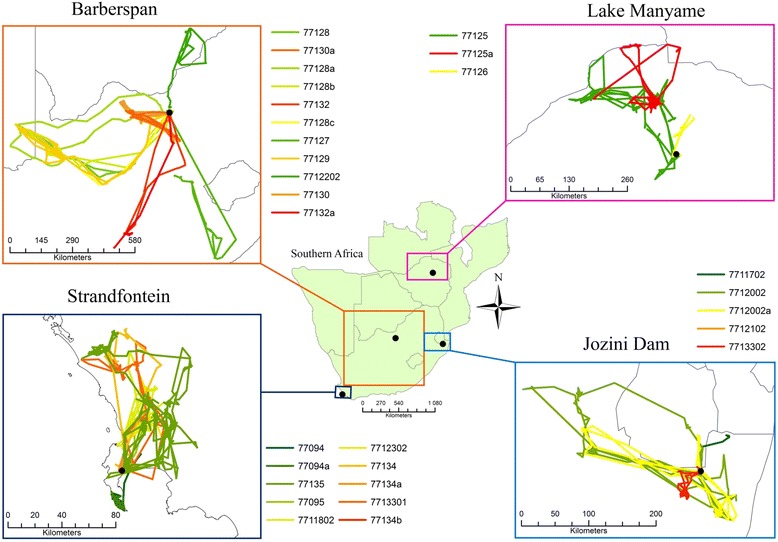
Maps showing the movement paths and capture sites of Egyptian Geese in southern Africa over the study period. Legend represents platform transmitter terminal identities of individuals at each site

**Fig. 6:**
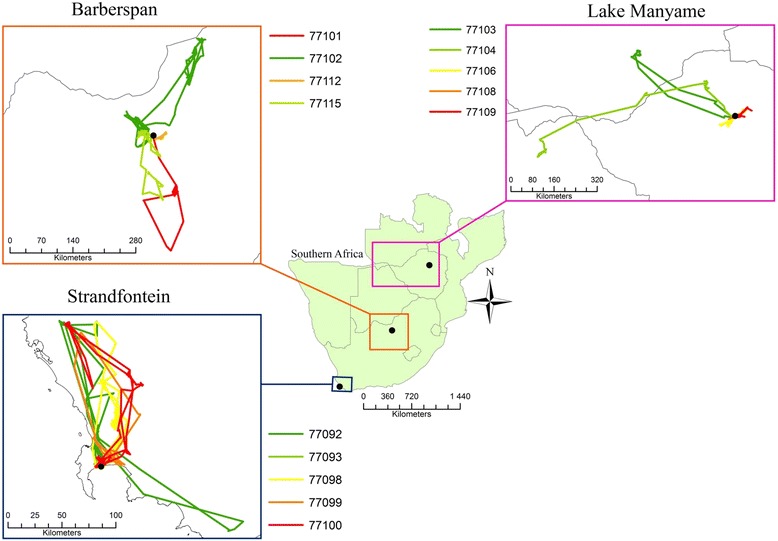
Maps showing the movement paths and capture sites of Red-billed Teal in southern Africa over the study period. Legend represents platform transmitter terminal identities of individuals at each site

**Fig. 7:**
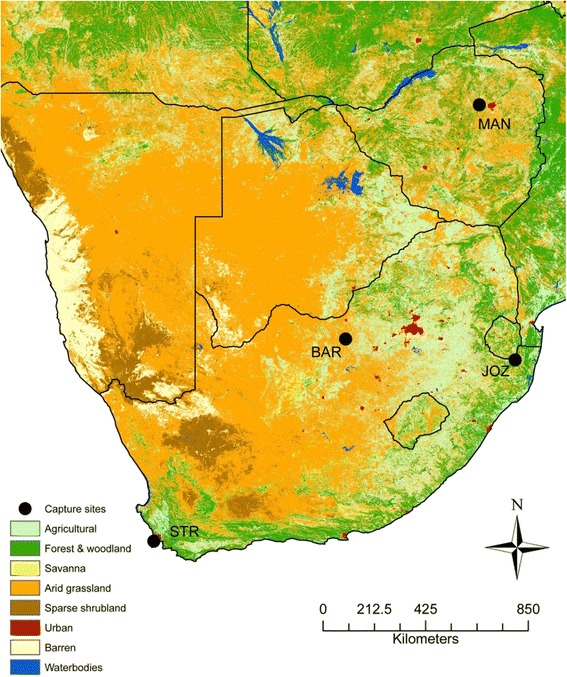
Land cover classes within the southern African landscape. Points represent capture sites for sampled individuals. BAR, Barberspan; JOZ, Jozini Dam; MAN, Lake Manyame; STR, Strandfontein
